# Exploring the Various Sources of Mortality Estimation in Ghana: A Scoping Review of Data Sources, Challenges, and Opportunities

**DOI:** 10.3390/healthcare13182331

**Published:** 2025-09-17

**Authors:** Regina Titi-Ofei, Hillary Kipruto, Dominic Atweam, Anthony Adofo Ofosu, Clementine Rossier

**Affiliations:** 1 Institute of Global Health, Faculty of Medicine, Université de Genève (UNIGE), 1202 Geneva, Switzerland; 2 Health Information Systems Unit, World Health Organization Regional Office for Africa, Brazzaville P.O. Box 06, Congo; kiprutoh@gmail.com; 3 Health Information Systems, World Health Organization, Accra P.O. Box M.B.142, Ghana; datweam@who.int; 4 Department of Health Policy, University of Health and Allied Sciences, Ho PMB 31, Ghana; anthony.ofosu65@gmail.com; 5 Institut de démographie et socioéconomie, Université de Genève (UNIGE), 1202 Geneva, Switzerland; clementine.rossier@unige.ch; 6 Institut National d’Etudes Démographiques, Aubervilliers CEDEX, 93322 Paris, France

**Keywords:** mortality estimation, civil registration and vital statistics, public health data

## Abstract

**Background:** Accurate estimation of mortality is essential for effective public health planning, policymaking, and monitoring of health interventions. In Ghana, multiple data sources are used to estimate mortality, including civil registration systems, household surveys, census data, and health and demographic surveillance systems. This scoping review explores the existing sources of mortality data in Ghana, examining their challenges and opportunities. **Methods:** Using Arksey and O’Malley’s framework, we identified peer-reviewed and grey literature from Ghana Health Service, Ministry of Health, Ghana Statistical Service, WHO, and the United Nations. We selected studies published between 2000 and 2024 that focused on mortality estimation in Ghana. Data was extracted and synthesized into key themes: data sources, challenges, and opportunities. **Results:** Six major data sources on mortality were identified: Civil Registration and Vital Statistics (CRVS), census data, Demographic and Health Surveys (DHS), Health and Demographic Surveillance Systems (HDSS), Facility-Based Health Information Systems (HMIS), modeled estimates from the Global Burden of Disease (GBD) and from the United Nations Department of Economic and Social Affairs (UN DESA). Key challenges include under-registration of deaths (CRVS and HMIS), recall bias (DHS, census), limited geographic coverage (HDSS), inconsistencies in cause-of-death classification (HMIS, HDSS), and lack of local geographic coverage (GBD, UN DESA, DHS). Nonetheless, benefits include longitudinal follow-up (HDSS), local coverage and ownership (CRVS, HMIS) and international comparability (GBD, UN DESA, DHS). **Conclusions:** Mortality estimation in Ghana is supported by diverse but fragmented data sources. Strengthening the CRVS and HMIS systems, integrating multiple data streams, standardizing methodologies, and enhancing institutional partnership are essential steps toward improving data quality and coverage. This review provides recommendations for improvement towards better quality estimations of mortality in Ghana.

## 1. Background

Accurate and comprehensive mortality data is essential for effective public health planning and achieving Sustainable Development Goal 3 (SDG 3). Mortality estimation enhances the ability to track trends, assess disparities, and evaluate public health interventions [1], and is crucial for strengthening countries’ health information ecosystems and ensuring data-driven health policies [2]. In several low-income contexts, including Ghana, estimations of mortality continue to be challenged due to limited functionality of civil registration and vital statistics systems (CRVS) and incomplete data from routine facility-based information systems (HMIS) [3,4]. Civil registration is the continuous, permanent, compulsory recording of the occurrence and characteristics of vital events such as birth and death. CRVS systems are responsible for the continuous and universal registration of vital events, such as births and deaths, along with cause-of-death information [5]. HMIS, on the other hand, compiles routine data from health facilities, including institutional deaths and causes of death. Both systems are central pillars of a country’s health information infrastructure and are vital for producing timely and disaggregated mortality data [3,6].

Improving mortality data systems is not only a national priority for several countries but also an overarching global priority. Mortality data is foundational for international health monitoring frameworks such as the Global Burden of Disease study, the United Nations Sustainable Development Goals (SDGs), and Universal Health Coverage (UHC) tracking. Inaccurate or incomplete mortality data undermines the ability of countries to allocate resources equitably, respond to emerging health threats, and benchmark progress [7]. Strengthening mortality surveillance in countries like Ghana therefore contributes to global health equity and accountability.

Overall, it is estimated that in Africa, less than 40% of countries have complete CRVS systems, and 90% of CRVS systems are assessed as nascent [8,9]. As a result, the availability of counts of deaths by age and sex from CRVS is largely unavailable and incomplete. These weaknesses in CRVS systems have led to reliance on less optimal approaches to estimating mortality in the country.

Despite Ghana’s significant economic transformation, transitioning from a low-income to a lower-middle-income country in 2010, and notable improvements in GDP growth, poverty reduction, and investments in infrastructure and social services, including health in the last decade, inequalities persist and the health system faces challenges in generating reliable mortality data, and a comprehensive evaluation of all-cause and cause-specific mortality estimations in Ghana has been lacking over the last decade [10,11,12].

This scoping review aims to map out the various data sources used for mortality estimation in Ghana, highlighting their challenges and opportunities to provide a clearer picture of mortality estimations in the country. The review identifies and describes various sources used to estimate mortality in Ghana, examining the challenges and strengths of each data source and providing recommendations for improving mortality data collection and estimation practices.

This study is motivated by the need to document the various existing data sources, assess their strengths, weaknesses, and opportunities for improvement, and identify pathways for optimizing their integration and use. In light of growing demands for timely and disaggregated mortality data to inform national and subnational policy decisions, the study also explores opportunities for methodological innovation—particularly approaches that enable the generation of timely all-cause mortality estimates at the subnational level.

## 2. Methods

We adopted Arksey and O’Malley’s scoping review framework, which includes identifying the research question, selecting relevant studies, charting the data, and collating, summarizing, and reporting the results [13]. It also aligns with the PRISMA-ScR (Preferred Reporting Items for Systematic Reviews and Meta-Analyses Extension for Scoping Reviews) guidelines. We conducted a comprehensive search of peer-reviewed articles from databases including PubMed, Web of Science, and Scopus, and consulted grey literature from the Ministry of Health (MoH), Ghana Health Service (GHS), Ghana Statistical Service (GSS), WHO, and the United Nations. An in-depth search strategy was used, combining keywords and Medical Subject Headings (MeSH). A sample search strategy is as follows: (“adult mortality” OR “adult death” OR “mortality estimation” OR “adult survival” OR “mortality rate”) [Outcome] AND (“Ghana”) [Setting] AND (“data source” OR “civil registration” OR “vital statistics” OR “demographic survey” OR “DHS” OR “HDSS” OR “census” OR “health information system” OR “HMIS” OR “hospital data” OR “verbal autopsy” OR “Global Burden of Disease” OR “GBD”) [Exposure/Data Source] AND English [lang]).

**Figure 1:**
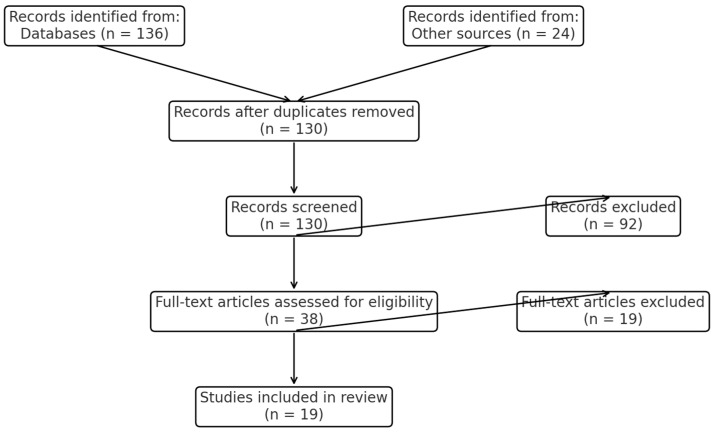
PRISMA flow diagram summarizing study selection process.

Figure 1 shows a PRISMA flow diagram summarizing the study selection process.

Two independent reviewers screened titles and abstracts against the review’s inclusion criteria. The full texts of chosen citations were retrieved, and a detailed assessment by the two independent reviewers was conducted against the inclusion criteria. The following inclusion criteria were used to guide the search and article review:

- Geographic Context: Ghana (national and subnational levels).
- Types of Sources: peer-reviewed articles, technical reports, institutional documents, government publications, and grey literature.
- Inclusion Criteria: studies or reports published between 2000 and 2024 in English that provide empirical or modeled estimates of adult mortality in Ghana.
- Language: English.
- Years of Publication: 2000–2024.

A standardized data extraction table was developed to systematically capture key information from each source. Extracted variables included data source type, geographic and temporal coverage, data collection periodicity, methodological strengths and limitations, and relevance for health policy and planning. This table facilitated comparative analysis and thematic synthesis of the included data sources.

The extracted information was organized into a comparative matrix enabling identification of cross-cutting patterns in data quality, representativeness, and policy utility. Table 1 in the Results Section presents this information.

The protocol for this scoping review has been registered in the Open Science Framework (OSF) database at https://doi.org/10.17605/OSF.IO/MQ6SP (accessed on 5 April 2025).

## 3. Results

Findings are described according to the various data sources for mortality estimation in Ghana.

### 3.1. Civil Registration and Vital Statistics (CRVS) in Ghana

CRVS systems are essential for the continuous and systematic recording of vital events such as births and deaths [14]. In Ghana, the CRVS system is underdeveloped, with limited coverage, particularly in rural areas. Despite these limitations, CRVS remains the cornerstone for reliable mortality statistics if adequately strengthened [15,16]. CRVS also provides a foundational mechanism for recording other key life events such as births, marriages, and divorces, offering numerous benefits for individuals and governments alike. In several countries, CRVS serves as the primary source of legal identity, enabling access to essential services such as education, healthcare, social protection, and voting rights. CRVS supports effective planning, policymaking, and resource allocation by providing timely and accurate population data. It strengthens public administration and enables monitoring of key development indicators [17].

While Ghana has made notable progress in birth registration, death registration coverage remains limited, estimated at just 23% nationally as of 2021 [15]. This gap critically impairs the country’s capacity to produce accurate mortality statistics, which are essential for evidence-based health and social planning.

Furthermore, Ghana’s CRVS system currently has limited technical and institutional capacity to generate disaggregated, cause-specific mortality data. The absence of medical certification of cause of death (MCCOD) in many communities, especially in rural areas, restricts the ability to estimate disease burden and track health outcomes. Most deaths occur outside health facilities, and where certification exists, it often lacks the WHO-recommended International Classification of Disease (ICD) structure.

Efforts to strengthen mortality estimation include pilot initiatives to improve MCCOD practices, integration of CRVS data with the country’s District Health Information Management System (DHIMS2), and capacity-building programs. Ghana’s CRVS Strategic Action Plan (2023–2030) explicitly targets improvements in mortality registration through expanded digitization, use of verbal autopsy tools in non-facility deaths, and enhanced coordination between ministries. However, sustained investments in training, infrastructure, and data use are needed to fully realize the CRVS system’s potential in supporting accurate and routine mortality estimation.

### 3.2. Census Data

The Population and Housing Census (PHC) in Ghana has played a central role in demographic data collection and national planning since independence in 1957. Conducted approximately every ten years, the census provides a comprehensive snapshot of the country’s population structure, housing conditions, and socio-economic characteristics. The first post-independence census was held in 1960, setting the foundation for future demographic work. Subsequent censuses—in 1970, 1984, 2000, 2010, and most recently in 2021—have evolved in scope and methodology. For instance, the 2000 census introduced more detailed modules on mortality, fertility, and housing; the 2010 census enhanced coverage and included household death data; and the 2021 census became the first fully digital operation, integrating real-time data capture and geospatial technologies.

In the context of mortality estimation, census data in Ghana has become increasingly valuable due to the limitations of the country’s civil registration and vital statistics (CRVS) system. Specifically, censuses provide indirect means to estimate mortality by including questions on household deaths in the past 12 months, parental survivorship (orphanhood), and sibling survival [18]. These allow researchers to estimate crude death rates, age-specific death rates, mortality ratios, and mortality differentials by sex, age, region, and socio-economic status [19]. The orphanhood method, for example, is widely used to estimate national-level mortality where information on the survival status of respondents’ parents serves as a proxy.

Despite these benefits, the use of census data for mortality estimation is not without challenges. Underreporting of deaths, recall errors, age misreporting, and the lack of cause-of-death data affect the accuracy and reliability of census-based mortality estimates [20]. Moreover, censuses typically only capture deaths that occurred within the 12 months prior to enumeration; the low frequency of censuses limits trend analysis. Moreover, the method based on parental survival and sibling survival can only yield national estimates, since the residence of these family members is not captured. Nonetheless, the national coverage, large sample sizes, and integration of socio-economic indicators make census data a crucial tool for examining mortality disparities and supporting public health interventions. In the absence of a complete CRVS system, census-based methods offer a practical and cost-effective approach to mortality estimation in Ghana, especially when triangulated with other data sources such as household surveys and health facility records [12].

### 3.3. Facility-Based Information Systems (HMIS) in Ghana

Hung et al. in their systematic review on the use of HMIS data for policy-making, establish that “HMIS data represent an underused source of data and should be made more available and further embraced by the research community in LMIC health systems” [21]. In Ghana, health facility data is collected and reported by different health facilities through a standardized health management information system (HMIS), using paper-based forms or reports. These reports are a source of patient data on mortality, morbidity, and additional health system indicators.

At the national level, routine health information systems have been explored in the generation of mortality estimates given their geographic scope and routine periodicity, as data is compiled on a routine basis (monthly) and covers all facilities [12]; however, estimates generated using the routine HMIS have been challenged in their precision and robustness, as about 40% of deaths in the country occur at the community level, and these have not been systematically incorporated in the estimates [3,22,23,24].

In Ghana, the HMIS system is based on The University of Oslo-supported District Health Information System version 2 (DHIS 2) and is referred to as DHIMS 2. The DHIMS 2 platform is implemented by the Ghana Health Service (GHS). It is a comprehensive system for remotely compiling data across different levels of a health system into a central storage point at the district level. Aggregation of data in DHIMS 2 is based on paper-based health facility registers that are completed by health workers in public facilities. DHIMS 2 captures data on health service provision, deaths and their causes, and allows for analyses at the district, regional, and sub-national level. As of 2013, the DHIMS 2 was accessible in 170 out of 216 districts with about 5163 registered users [25]. DHIMS 2 tracks monthly counts of a wide range of health service delivery indicators, including immunization, family planning, reproductive, maternal, and child health indicators, and demographic indicators, including facility-reported births and deaths. Given that DHIMS 2 includes data from all reporting health facilities, these indicators are reported as counts.

A study from Owusu et al. (2021) demonstrated the use of the routine facility-based information system for mortality estimation in Ghana [12]. They derived estimates for all-cause and cause-specific mortality using the District Health Management Information System. The method, aligned with the Ghana Health Service Standard Operating Procedures for mortality estimations, generated estimates of facility-based mortality by dividing all institutional deaths by all institutional admissions per 1000 [(all institutional deaths/all institutional admission) x 000]. The study documented a major limitation in the estimates due to lack of incorporation of deaths from the community.

### 3.4. Health and Demographic Surveillance System in Ghana

Health and Demographic Surveillance Systems (HDSS) have been posited as a gold standard for mortality estimation in low-resource settings in the absence of CRVS. The INDEPTH network in the early 2010s operated over 50 HDSS member sites across various low-income settings, with 48 of them in Sub-Saharan Africa [26]. HDSS utilizes a sentinel surveillance mechanism to follow a locally defined population (the population on a fixed territory) through repeated household visits. These individuals are followed across their life span, entering the sentinel surveillance cohort at the initial census or through birth or immigration and exiting through emigration or death. Surveillance of this population involves household visits, where interviewers review the list of household members who were present at the previous visit and capture key demographic data, including information on births, deaths, migration, civil registration for births, and data on identified health indicators. The design of HDSS sites makes them a significantly comprehensive source of civil registration and vital statistics data, with limited likelihood of not capturing key data on the target population; they are, however, limited in their geographic coverage given that they are limited usually to one or two health districts [27,28,29]. Moreover, some research has documented evidence that people living within the HDSS catchment areas have a greater demand for or use of health services, consequently leading to better health outcomes compared to the equivalent non-HDSS populations [30,31]. For example, an analysis from the Butajira HDSS in Ethiopia showed that antenatal care coverage was higher among the HDSS population that for the population outside of surveillance.

Verbal autopsy (VA) is a widely used method within Health and Demographic Surveillance Systems (HDSS) to determine the probable causes of death in settings where civil registration and vital statistics (CRVS) systems are weak or incomplete [32]. In HDSS, trained fieldworkers conduct interviews with close relatives or caregivers of the deceased, using a structured questionnaire to capture details about symptoms, medical history, and the circumstances leading up to death [33]. This approach enables the generation of cause-specific mortality estimates and supports public health planning by providing evidence on the burden of diseases. In Ghana, several HDSS sites (the Navrongo, Kintampo, and Dodowa health research centers) have routinely implemented verbal autopsy as part of their mortality surveillance efforts [34]. These sites continuously monitor population dynamics, including births, deaths, and migrations, and link this information to VA-generated cause-of-death data. Despite its strengths, verbal autopsy is not without limitations. It can be affected by recall bias, misclassification—particularly for non-specific symptoms and chronic conditions—and cultural sensitivities that may limit accurate reporting of symptoms before death [35]. Nevertheless, VA within HDSS provides cost-effective approaches to understanding cause-specific mortality at the local level.

The section below provides an overview of the three HDSS sites in Ghana.

- Navrongo HDSS—Upper East Region

The Navrongo Health and Demographic Surveillance Site (HDSS), established in 1992 in the Kassena-Nankana district of Ghana’s Upper East Region, is the country’s oldest and most well-established HDSS site. It covers two districts: Kassena-Nankana East Municipality and Kassena-Nankana West District in the Upper East Region. Managed by the Navrongo Health Research Centre (NHRC) under the Ghana Health Service, it monitors a population of approximately 160,000 individuals through routine household visits to track vital events such as births, deaths, and migrations [34]. The site played a pioneering role in the development and testing of the Community-based Health Planning and Services (CHPS) initiative, which was later scaled nationally to improve primary healthcare delivery. Research at the Navrongo HDSS focuses on maternal and child health, fertility and mortality trends, malaria control, and vaccine trials. It also conducts verbal autopsies to determine causes of death, contributing valuable data for public health policy and intervention strategies in rural northern Ghana [36].

- Kintampo HDSS—Bono East Region

The Kintampo Health and Demographic Surveillance Site (HDSS), established in 2003, operates in the Kintampo North and South districts as well as four other districts in the Bono East Region and two districts in the Ahafo Region, covering a population of around 150,000 people across approximately 30,000 households. It is managed by the Kintampo Health Research Centre (KHRC) under the Ghana Health Service and conducts regular household visits to record demographic events such as births, deaths, and migrations. The Kintampo HDSS is known for its work on maternal and neonatal health, vaccine trials (including malaria vaccines), and emerging non-communicable diseases [37]. The site also collects detailed socioeconomic data, supporting multi-sectoral analysis and informing national health policy. The Kintampo HDSS is known for its detailed social and economic data collection and has hosted large-scale randomized controlled trials. It plays a key role in vaccine monitoring and implementation research.

- Dodowa HDSS—Greater Accra Region

The Dodowa Health and Demographic Surveillance Site (HDSS), initiated in 2005, is located in the Shai-Osudoku and Ningo-Prampram districts of the Greater Accra Region and is managed by the Dodowa Health Research Centre (DHRC). It monitors a population of approximately 120,000 individuals and focuses on the health implications of urbanization and rural-urban transition. The site collects routine data on births, deaths, and migration and conducts verbal autopsies to determine causes of death. Research themes include reproductive and child health, nutrition, school health programs, and health systems strengthening [38]. As a peri-urban HDSS, Dodowa provides critical insights into the shifting disease burden and health service needs in rapidly changing communities near the capital city.

### 3.5. Sample-Based Population Surveys in Ghana—Demographic and Health Survey

Ghana has participated in Demographic and Health Surveys (DHS) over the past thirty (30) years. The Ghana Demographic and Health Survey is a nationally representative cross-sectional survey of population health and nutrition. The DHS follows a multi-stage sampling procedure and is a nationally representative dataset for monitoring population health trends. The DHS was conducted in 1988, 1993, 1998, 2003, 2008, 2014, and 2022. The 1988 Ghana Demographic and Health Survey (GDHS) established baseline data on fertility, family planning, and maternal and child health, offering insights into the country’s demographic and health status. The 2022 GDHS is the most recent survey conducted by the Ghana Statistical Service, offering estimations on key indicators related to fertility, family planning, childhood mortality, maternal and child health, nutrition, HIV prevention knowledge, and other health issues. The Demographic and Health Surveys (DHS) have been instrumental in estimating mortality rates in Ghana, providing comprehensive data that informs health policies and programs. Specific to adult mortality, the 2022 Ghana DHS included questions on sibling survival to estimate adult mortality rates [39]. This approach involves respondents providing information about their siblings’ survival, which is then used to estimate adult mortality rates [18]. Such data are vital for understanding the broader health landscape and informing interventions targeting adult populations. However, only national-level estimates are possible, since the residence of siblings is not tracked. DHS has remained the mainstay of large-scale sample population-based surveying in Ghana.

### 3.6. The United Nations Population Division—Age-Sex Specific Mortality Estimation (UN DESA)

In the absence of reliable morality estimations from local data sources, the United Nations Division of Population Affairs (UN DESA) generates modelled estimates of age-sex-specific mortality across all countries, including projections into the future. These indirect mortality estimations, applying a Bayesian Hierarchical Model Framework [40], provide key insights and allow for the availability of mortality data in Ghana, despite the challenges with the various locally available data sources.

The UN DESA Bayesian Hierarchical Model (BHM) allows the incorporation of multiple data sources, taking into account the uncertainty and biases in the data [41].

The model adopts a logit scale for mortality estimation, constraining probabilities between 0 and 1. The BHM framework allows observations as well as covariates such as under-five mortality rates, prevalence of HIV infection, and coverage of antiretroviral therapy (ART) to be incorporated in the model, using a flexible non-linear regression structure that captures temporal trends and accounts for data heterogeneity.

In the generation of country-specific mortality estimations, a separation of the modelling methods is made for non-HIV and HIV countries. In the estimation, Ghana is classified as a non-HIV country. The methods proposed by UN DESA also make a distinction in the modeling methods for countries with differentiated coverage and quality levels for mortality data. The three categories established are as follows:

1. Countries that possess comprehensive and reliable mortality data, typically sourced from civil registration systems. These are generally high-income or upper-middle-income countries with well-established administrative mechanisms for recording births and deaths.
2. Countries that lack high-quality civil registration systems but have alternative data sources such as household surveys, population censuses, or modeled estimates with sufficient temporal coverage. These countries, often low- or middle-income, rely on periodic data collection efforts rather than continuous administrative records.
3. Countries with very limited data or no information on adult mortality.

In addition, there is a transversal distinction made for countries with high HIV prevalence.

The model also accounts for both sampling and non-sampling sources of error. Sampling errors originate from the structure of survey designs or elements of randomness characteristic of data from administrative datasets, while non-sampling errors result from issues such as non-response, recall bias, or mistakes during data entry. These error types are explicitly modeled to generate reliable uncertainty bounds around mortality estimates. This rigorous approach enhances the accuracy of mortality estimations, offering valuable data for demographic research and informing evidence-based policymaking.

Since the age- and sex-specific mortality estimates in Ghana are derived from survey-based (non-CRVS) sources, the associated sampling errors are computed from the underlying microdata. In instances where sampling errors are unavailable, they are estimated by imputing the median sampling error observed within each age-sex group and the corresponding recall period between the event and the survey interview. These estimates are then refined using simulated values drawn from a normal distribution, with the mean set to the observed mortality and the standard deviation matching the computed sampling error. The standard deviation of these simulations—transformed to the logit scale—serves as the final estimate of the sampling error for non-CRVS data.

As stated earlier, Ghana fits into the category of countries with low HIV prevalence but with limited availability of vital statistics data, given the current coverage of about 20% [15]. Thus, the methods described by Chao et al. for the generation of age–sex-specific mortality estimations are applied to Ghana for the generation of the mortality estimations. While useful for global comparisons, modelled estimates from UN DESA rely heavily on the quality of input data and assumptions that may not reflect local realities [42]. Moreover, they are available only at the national level.

### 3.7. The Global Burden of Disease—Mortality Estimation

Another indirect mortality estimation data source that is globally generated is the Global Burden of Disease (GBD) study, led by the Institute for Health Metrics and Evaluation (IHME). The GBD provides comprehensive and comparable estimates of mortality and morbidity across countries, including Ghana, which is a context in which the civil registration and vital statistics (CRVS) systems are incomplete. The GBD mortality estimation methodology integrates a wide range of data sources—such as household surveys (e.g., DHS), population censuses, sample registration systems, health facility records, and verbal autopsies—and applies advanced statistical modeling techniques to generate robust estimates. Specifically, the estimation adopts Cause of Death Ensemble modeling (CODEm) and spatiotemporal Gaussian process regression to estimate age-, sex-, cause-, and location-specific mortality [43]. These models adjust for known biases, underreporting, and inconsistencies across data sources. In the context of Ghana, GBD estimates provide valuable insights into mortality patterns and causes of death, particularly for non-communicable diseases, injuries, and infectious diseases affecting the working-age population. Although the estimates are model-based and may not always capture recent epidemiological shifts or local variations, the GBD serves as a critical tool in the national mortality data ecosystem. It informs health policy, priority setting, and tracking of progress toward national and global health targets.

## 4. Discussion

Reliable mortality estimation is crucial for health system planning and evaluation. In Ghana, several mortality data sources offer some insights, but all are constrained by methodological and infrastructural limitations.

While CRVS remains the gold standard, its underperformance necessitates reliance on multiple alternative sources. DHS and census data offer national-level snapshots, whereas HDSS provides in-depth, localized insights, despite its limitation of not being representative at the national level. Similarly, mortality counts from HMIS and CRVS are challenged given the high risk of underreporting and, for HMIS, the likelihood of missing the capture of deaths occurring at the household and community level.

The challenges associated with each data source underscore the need for systemic reform. The limitations across these data systems are reflective of broader infrastructural and administrative weaknesses, including insufficient funding, lack of trained personnel, and poor interoperability between government agencies. Targeted investments to strengthen CRVS and HMIS, including infrastructural reforms through digitization, rural outreach, and public education campaigns, could significantly improve data completeness and timeliness.

**Figure 2:**
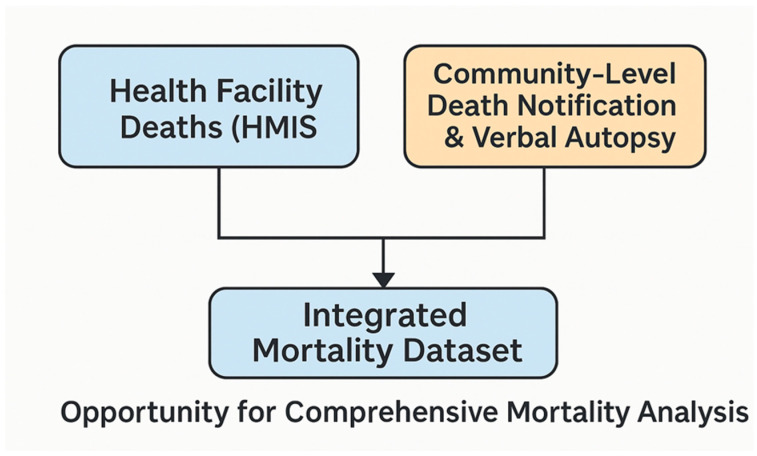
Conceptual schema for integrating community and facility-based mortality data for comprehensive mortality estimation. Source: Authors’ conceptualization.

Furthermore, HMIS captured deaths, while readily available, and operationally important for health system management, usually underrepresent deaths that occur outside health facilities. We propose that methodological options for integrating facility-based and community-based deaths be explored in the research to generate a more robust and integrated picture of deaths. Subsequent studies could explore a methodological framework for mortality data estimation at the local or district level, which integrates multiple data sources and employs robust statistical techniques. The methodological frame will take into account health facility and community-based deaths at the district level, allowing for a more refined understanding of mortality patterns. Currently, methods to achieve such robust estimates, where facility and community deaths are combined at the local level, are sparse in the literature. Early work on such frameworks for combining facility and community deaths for all-cause mortality estimation in Sub-Saharan Africa has commenced, with the work of Issah et al. [44]. In Tanzania, a study by Whiting et al. has generated mortality estimates from community- and facility-based data sources using this approach. In their paper, data from sentinel vital registration (SVR) with verbal autopsy (VA) were used to determine the mortality burden at the community level in two areas of the United Republic of Tanzania. In their analysis, proportional cause-specific mortality structures obtained from health facility information systems were applied to counts of deaths obtained from the community-based sentinel system to generate robust and integrated mortality estimates [45]. Figure 2 provides a conceptual schema for integrating community and facility-based mortality data for comprehensive mortality estimation.

In contrast, household surveys such as the DHS, while nationally representative, are constrained by their periodic nature and reliance on respondent recall. These constraints introduce potential bias, particularly when respondents are required to remember events that occurred several years earlier. Innovations in survey design, including shortened recall periods, improved question phrasing, and integration with administrative data, could enhance data quality. In addition to these, in recent times, the DHS is also threatened by funding issues because of the withdrawal of its major funder, USAID, from the development financing landscape. This calls for other external funders to explore opportunities to support the DHS, which remains a key pillar in population-based health statistics and demography.

In Ghana, the decennial national housing and population census offers an opportunity to collect comprehensive demographic information, including mortality-related indicators. However, challenges such as outdated sampling frames, enumeration errors, inconsistent quality control, and logistical limitations, especially in remote areas, undermine the reliability of census-derived mortality data. Strengthening census processes would require enhanced inter-agency coordination, greater investment in digital enumeration tools, and capacity-building for field staff. There is also a growing opportunity to integrate census data with administrative records and geospatial technologies to improve accuracy and enable small-area mortality estimation. Institutionalizing post-enumeration surveys and improving public trust through transparent processes can further strengthen the credibility and utility of census data in mortality estimation.

The role of HDSS is particularly noteworthy. HDSS sites, though limited in coverage, generate rich, continuous datasets that include verbal autopsy data and socio-demographic indicators. Findings from HDSS sites have influenced national health policy and provided valuable insights into mortality trends. There is a compelling case for expanding the HDSS network across diverse ecological zones in Ghana, ensuring broader representativeness and regional equity in mortality estimation. As the country plans for scale-up, existing HDSS mortality data can also be leveraged in the generation of model estimations of mortality based on district-level health facility mortality data obtained through the district health information systems.

Global modeled estimates from the UN DESA or Global Burden of Disease offer the advantage of cross-country comparability and trend monitoring. However, these models depend heavily on assumptions and extrapolated data from other low-income countries. Their utility in Ghana hinges on the inclusion of high-quality local inputs. Strengthening national data systems not only improves internal policy and planning but also enhances the reliability of international models.

An integrated approach to mortality estimation—one that leverages existing data to promote better quality and more complete CRVS and HMIS—offers the most promising path forward. This requires political will, cross-sector collaboration, and capacity-building at all levels of the health information system. Cross-sector collaboration could include the establishment of communities of practice comprised of researchers, policymakers, and health workers to collectively deliberate and propose concrete solutions for implementing such integrated mortality estimation approaches and institutionalizing them. At the district level, district health officers can ensure compliance with the generation of such mortality statistics to be reported to the national level on a routine basis. Enhanced partnership between government institutions such as the Ghana Statistical Service, Ghana Health Service, and academia will be important to achieve this.

Furthermore, this study holds critical implications for public policy and national decision-making in Ghana. By systematically mapping the strengths and limitations of each mortality data source, the review provides a valuable evidence base to inform government efforts aimed at enhancing the quality, completeness, and use of mortality data for health planning and performance monitoring. The findings directly support priorities outlined in the Ghana Health Sector Medium-Term Development Plan 2022–2025, which emphasizes the need for improved data systems to inform equitable service delivery, especially in underserved regions [46]. It also reinforces the importance of implementing Ghana’s Civil Registration and Vital Statistics Strategic Action Plan (2023–2030) and the Ghana Health Information System Strategic Plan (2022–2025), which calls for expanded digitization, improved cause-of-death certification, and the institutionalization of verbal autopsy for deaths occurring outside health facilities and promotes data interoperability across systems [47].

In terms of study limitations, this scoping review did not include a formal quality appraisal of the included sources. While such an assessment can enhance interpretability, it was not undertaken due to the already limited availability of the literature, both peer-reviewed and grey, on mortality estimation sources in Ghana. Given this sparse evidence base, the primary aim of the review was to comprehensively map and synthesize all relevant sources to provide an overview of the current landscape. Future research could build on this foundational mapping by conducting more detailed and systematic quality appraisals of specific data systems and mortality estimation methods.

## 5. Conclusions

Reliable data on who dies, when, where, and from what cause is a cornerstone of equitable health systems, optimal resource allocation, and evidence-based policy [7]. This scoping review set out to explore and map the various data sources used to estimate mortality in Ghana, with a particular focus on identifying their respective strengths and limitations. It has examined how different sources, including CRVS, household surveys, censuses, HDSS, health facility records, and global modeled estimates, contribute to understanding mortality levels and patterns in the country. By analyzing the methodological challenges and practical benefits of each data type, the review provides a comprehensive overview of the current state of mortality estimation and informs efforts for strengthening the country’s health information system to enable better mortality estimation capacities.

Ghana has a great opportunity to improve mortality estimation. By addressing these systemic weaknesses, the country can move towards a more resilient and responsive mortality surveillance system. This, in turn, will support data-driven policymaking for equitable health outcomes and achieving national and global health targets.

## Figures and Tables

**Table 1:** Comparative overview of mortality estimation data sources in Ghana, including coverage, periodicity, strengths, limitations, and policy utility.

Data Source	Coverage	Periodicity	Strengths	Limitations	Policy Utility
**Civil Registration and Vital Statistics (CRVS)**	National and Sub-National (limited, ~23% death registration in Ghana)	Continuous	Legal basis for registration, potential for comprehensive cause-of-death data, integration with national ID systems	Low coverage, weak cause-of-death certification, underreporting in rural areas	Core for national statistics and planning if strengthened
**Health Management Information System (HMIS)**	National and Sub-national- Health facilities	Monthly	Routine reporting, real-time data aggregation, district-level disaggregation	Excludes community deaths, data quality concerns, underreporting	Useful for operational monitoring, not fully representative of mortality estimation
**Census**	National	Decennial	Large sample, national representativeness, socio-demographic context	Recall bias, age misreporting, no cause-of-death data, infrequent	Supports indirect mortality estimation and demographic planning
**Demographic and Health Survey (DHS)**	National sample	Every 5–7 years	Sibling survival data, national estimates, standardized methods	Recall bias, no subnational estimates for adult mortality, infrequent	Monitoring health trends, supporting model-based estimates
**Health and Demographic Surveillance Systems (HDSS)**	Sub-national- Navrongo HDSS: Kassena-Nankana East and West (Upper East Region); Kintampo HDSS: Kintampo North and South, Nkoranza North and South, Techiman North and South (Bono East Region), Asutifi North and South (Ahafo Region); Dodowa HDSS: Shai-Osudoku and Ningo-Prampram (Greater Accra Region)	Continuous	Longitudinal tracking, verbal autopsy, high completeness in catchment area	Limited geographic scope, potential Hawthorne effect	Valuable for local-level analysis, research, and model calibration
**UN DESA Modeled Estimates**	National	Biennial/Annual	Bayesian modeling framework incorporating multiple data sources (e.g., surveys, censuses) across time points; adjusts for bias and uncertainty; enables cross-country comparability and time trend analysis	Dependent on quality of input data, not locally owned	Used for international reporting and global monitoring frameworks
**Global Burden of Disease (GBD)**	National	Annual	Advanced statistical modeling, cause-specific mortality	Not real-time, estimates not always locally validated	Supports global health tracking, strategic policy inputs

## Data Availability

Not applicable.

## References

[1] AbouZahr C., Boerma T. (2005). Health information systems: The foundations of public health. Bull. World Health Organ..

[2] Phillips D.E., AbouZahr C., Lopez A.D., Mikkelsen L., De Savigny D., Lozano R., Wilmoth J., Setel P.W. (2015). Are well functioning civil registration and vital statistics systems associated with better health outcomes?. Lancet.

[3] Birabwa C., Banke-Thomas A., Semaan A., van Olmen J., Kananura R.M., Arinaitwe E.S., Waiswa P., Beňová L. (2024). The quality of routine data for measuring facility-based maternal mortality in public and private health facilities in Kampala City, Uganda. Popul. Health Metr..

[4] Wang H., Naghavi M., Allen C., Barber R.M., Bhutta Z.A., Carter A., Casey D.C., Charlson F.J., Chen A.Z., Coates M.M. (2016). Global, regional, and national life expectancy, all-cause mortality, and cause-specific mortality for 249 causes of death, 1980–2015: A systematic analysis for the Global Burden of Disease Study 2015. Lancet.

[5] United Nations Demographic and Social Statistics (2021). Civil Registration and Vital Statistics.

[6] Hong T.T., Hoa N.P., Walker S.M., Hill P.S., Rao C. (2018). Completeness and reliability of mortality data in Viet Nam: Implications for the national routine health management information system. PLoS ONE.

[7] Jackson D., Wenz K., Muniz M., Abouzahr C., Schmider A., Bratschi M.W., Braschi M.W., Kassam N., Diaz T., Mwamba R. (2018). Civil registration and vital statistics in health systems. Bull. World Health Organ..

[8] World Health Organization (2020). SCORE for Health Data Technical Package: Global Report on Health Data Systems and Capacity, 2020.

[9] United Nations Economic Commission for Africa Africa Has Made Progress in Civil Registration and Vital Statistics but Much Remains to Be Done. 14 October 2019. https://archive.uneca.org/stories/africa-has-made-progress-civil-registration-and-vital-statistics-much-remains-be-done.

[10] Agyemang C., Attah-Adjepong G., Owusu-Dabo E., De-Graft Aikins A., Addo J., Edusei A.K., Nkum B.C., Ogedegbe G. (2012). Stroke in Ashanti region of Ghana. Ghana Med. J..

[11] Lee Q.Y., Odoi A.T., Opare-Addo H., Dassah E.T. (2012). Maternal mortality in Ghana: A hospital-based review. Acta Obstet. Gynecol. Scand..

[12] Owusu A.Y., Kushitor S.B., Ofosu A.A., Kushitor M.K., Ayi A., Awoonor-Williams J.K. (2021). Institutional mortality rate and cause of death at health facilities in Ghana between 2014 and 2018. PLoS ONE.

[13] Arksey H., O’Malley L. (2005). Scoping studies: Towards a methodological framework. Int. J. Soc. Res. Methodol..

[14] Suthar A.B., Khalifa A., Yin S., Wenz K., Fat D.M., Mills S.L., Nichols E., AbouZahr C., Mrkic S. (2019). Evaluation of approaches to strengthen civil registration and vital statistics systems: A systematic review and synthesis of policies in 25 countries. PLoS Med..

[15] Ghana Statistical Service (2022). Civil Registration and Vital Statistics System in Ghana: Report on the Comprehensive Assessment.

[16] Mikkelsen L., Phillips D.E., AbouZahr C., Setel P.W., de Savigny D., Lozano R., Lopez A.D. (2015). A global assessment of civil registration and vital statistics systems: Monitoring data quality and progress. Lancet.

[17] World Bank Group, World Health Organization (2014). Global Civil Registration and Vital Statistics Scaling up Investment Plan 2015–2024.

[18] Masquelier B., Menashe-Oren A., Reniers G., Timæus I.M. (2024). A new method for estimating recent adult mortality from summary sibling histories. Popul. Health Metr..

[19] Obermeyer Z., Rajaratnam J.K., Park C.H., Gakidou E., Hogan M.C., Lopez A.D., Murray C.J.L. (2010). Measuring Adult Mortality Using Sibling Survival: A New Analytical Method and New Results for 44 Countries, 1974–2006. PLoS Med..

[20] Schmertmann C., Lanza Queiroz B., Gonzaga M. (2024). Data errors in mortality estimation: Formal demographic analysis of under-registration, under-enumeration, and age misreporting. Demogr. Res..

[21] Hung Y.W., Hoxha K., Irwin B.R., Law M.R., Grépin K.A. (2020). Using routine health information data for research in low- and middle-income countries: A systematic review. BMC Health Serv. Res..

[22] Adair T. (2021). Who dies where? Estimating the percentage of deaths that occur at home. BMJ Glob. Health.

[23] Akakpo P.K., Imbeah E.G., Agyarko-Wiredu F., Awlavi K., Baah-Amoh K., Derkyi-Kwarteng L. (2020). Community Causes of Death in the Central Region of Ghana, the Missing Piece in Mortality Data. Adv. Public Health.

[24] Poulin D., Nimo G., Royal D., Joseph P.V., Nimo T., Nimo T., Sarkodee K., Attipoe-Dorcoo S. (2024). Infant mortality in Ghana: Investing in health care infrastructure and systems. Health Aff. Sch..

[25] Odei-Lartey E.O., Prah R.K.D., Anane E.A., Danwonno H., Gyaase S., Oppong F.B., Afenyadu G., Asante K.P. (2020). Utilization of the national cluster of district health information system for health service decision-making at the district, sub-district and community levels in selected districts of the Brong Ahafo region in Ghana. BMC Health Serv. Res..

[26] Sankoh O., Byass P. (2012). The INDEPTH Network: Filling vital gaps in global epidemiology. Int. J. Epidemiol..

[27] Pison G. (2005). Population observatories as sources of information on mortality in developing countries. Demogr. Res..

[28] Rossier C., Schoumaker B., Delaunay V., Beguy D., Jain A., Bangha M., Aregay A., Beck B., Derra K., Millogo M. (2020). Adolescent Fertility Is Lower than Expected in Rural Areas: Results from 10 African HDSS. Stud. Fam. Plan..

[29] Streatfield P.K., Khan W.A., Bhuiya A., Alam N., Sié A., Soura A.B., Bonfoh B., Ngoran E.K., Weldearegawi B., Jasseh M. (2014). Cause-specific mortality in Africa and Asia: Evidence from INDEPTH health and demographic surveillance system sites. Glob. Health Action.

[30] Afework M.F., Gebregiorgis S.H., Roro M.A., Lemma A.M., Ahmed S. (2014). Do Health and Demographic Surveillance Systems benefit local populations? Maternal care utilisation in Butajira HDSS, Ethiopia. Glob. Health Action.

[31] Deribew A., Ojal J., Karia B., Bauni E., Oteinde M. (2016). Under-five mortality rate variation between the Health and Demographic Surveillance System (HDSS) and Demographic and Health Survey (DHS) approaches. BMC Public Health.

[32] Baiden F., Bawah A., Biai S., Binka F., Boerma T., Byass P., Chandramohan D., Chatterji S., Engmann C., Greet D. (2007). Setting international standards for verbal autopsy. Bull. World Health Organ..

[33] Byass P., Chandramohan D., Clark S.J., D’Ambruoso L., Fottrell E., Graham W.J., Herbst A.J., Hodgson A., Hounton S., Kahn K. (2012). Strengthening standardised interpretation of verbal autopsy data: The new InterVA-4 tool. Glob. Health Action.

[34] Oduro A.R., Wak G., Azongo D., Debpuur C., Wontuo P., Kondayire F., Welaga P., Bawah A., Nazzar A., Williams J. (2012). Profile of the Navrongo Health and Demographic Surveillance System. Int. J. Epidemiol..

[35] Nichols E.K., Ragunanthan N.W., Ragunanthan B., Gebrehiwet H., Kamara K. (2019). A systematic review of vital events tracking by community health agents. Glob. Health Action.

[36] Awoonor-Williams J.K., Sory E.K., Nyonator F.K., Phillips J.F., Wang C., Schmitt M.L. (2013). Lessons learned from scaling up a community-based health program in the Upper East Region of northern Ghana. Glob. Health Sci. Pract..

[37] Owusu-Agyei S., Nettey O.E.A., Zandoh C., Sulemana A., Adda R., Amenga-Etego S., Mbacke C. (2012). Demographic patterns and trends in Central Ghana: Baseline indicators from the Kintampo Health and Demographic Surveillance System. Glob. Health Action.

[38] Gyapong M., Sarpong D., Awini E., Manyeh A.K., Tei D., Odonkor G., Agyepong I.A., Mattah P., Wontuo P., Attaa-Pomaa M. (2013). Profile: The Dodowa HDSS. Int. J. Epidemiol..

[39] Bhalotra S. (2008). Sibling-Linked Data in the Demographic and Health Surveys. Econ. Polit. Wkly..

[40] Chao F., Williams I., Zeifman L., Gerland P. (2024). Estimating Age-Sex-Specific Adult Mortality in the World Population Prospects: A Bayesian Modelling Approach.

[41] Bijak J., Bryant J. (2016). Bayesian demography 250 years after Bayes. Popul. Stud..

[42] Vos T., Lim S.S., Abbafati C., Abbas K.M., Abbasi M., Abbasifard M., Abbasi-Kangevari M., Abbastabar H., Abd-Allah F., Abdelalim A. (2020). Global burden of 369 diseases and injuries in 204 countries and territories, 1990–2019: A systematic analysis for the Global Burden of Disease Study 2019. Lancet.

[43] Naghavi M., Makela S., Foreman K., O’Brien J., Pourmalek F., Lozano R. (2010). Algorithms for enhancing public health utility of national causes-of-death data. Popul. Health Metr..

[44] Issah M., Mug’atu J., Kipruto H.K. (2023). Weighted Bayesian Fractional Regression Model for Estimating Community Deaths in Africa. Int. J. Sci. Res. Eng. Dev..

[45] Whiting D.R., Setel P.W., Chandramohan D., Wolfson L.J., Hemed Y., Lopez A.D. (2006). Estimating cause-specific mortality from community- and facility-based data sources in the United Republic of Tanzania: Options and implications for mortality burden estimates. Bull. World Health Organ..

[46] Ministry of Health (2021). Ghana Health Sector Medium Term Development Plan 2022–2025.

[47] Ministry of Health (2022). Ghana Health Information System Strategic Plan.

